# Sarcopenia indicate poor survival in patients undergoing transarterial chemoembolization (TACE) for hepatic malignancies

**DOI:** 10.1007/s00432-022-04519-8

**Published:** 2023-01-23

**Authors:** Sven H. Loosen, Markus S. Jördens, Berenike Schoon, Gerald Antoch, Tom Luedde, Peter Minko, Christina Loberg, Christoph Roderburg

**Affiliations:** 1 Department of Gastroenterology, Hepatology and Infectious Diseases, University Hospital Düsseldorf, Medical Faculty of Heinrich Heine University Düsseldorf, Moorenstraße 5, 40225 Düsseldorf, Germany; 2 Department of Diagnostic and Interventional Radiology, University Hospital Düsseldorf, Medical Faculty of Heinrich Heine University Düsseldorf, Moorenstraße 5, 40225 Düsseldorf, Germany

**Keywords:** HCC; TACE, Cancer, Body composition, Computed tomography

## Abstract

**Background:**

Patient selection for transarterial chemoembolization (TACE) has remained challenging. Currently used markers mainly reflect liver function and turned out as less reliable in larger clinical trials. The patients´ body composition has been linked with patient outcome in different cancers. Now, we analyzed the function of different parameters of the patient’s body composition as prognostic and/ or predictive parameters in patients that received TACE.

**Methods:**

CT scans were used to assess five parameters of the individual body composition (skeletal muscle index (SMI), median muscular attenuation (MMA), bone mineral density (BMD) as well as the visceral and subcutaneous fat area) in 89 patients undergoing TACE. Results were correlated with tumor response to TACE and outcome of patients.

**Results:**

SMI and visceral fat area were significantly higher in male patients and among patients undergoing TACE for HCC compared to patients with liver metastases. While all parameters of the body composition did not predict response to TACE, patients with an SMI below the ideal cutoff value of 37.76 cm^2^/m^2^ had a significantly reduced long-term outcome with a median overall survival of 404 days compared to 1321 days for patients with a high SMI. Moreover, the pre-interventional SMI turned out as an independent prognostic factor in a multivariate Cox regression model including clinicopathological parameters and laboratory markers of organ dysfunction and systemic inflammation (HR: 0.899, 95% CI 0.827–0.979, *p* = 0.014).

**Conclusion:**

The pre-interventional SMI represents an independent prognostic factor for overall survival following TACE. Assessment of the individual body composition using routine CT scan might help to identify the ideal patients for TACE.

**Supplementary Information:**

The online version contains supplementary material available at 10.1007/s00432-022-04519-8.

## Introduction

Treatment algorithms for both primary (hepatocellular carcinoma) and secondary liver tumors (metastases) have changed significantly in recent years (Galle et al. 2018). In addition to significant improvements in system therapies, improved techniques for loco-regional interventions offer new therapeutic options for many patients (Galle et al. 2018; Bruix et al. 2015). In this context, transarterial chemoembolization (TACE) has become a novel standard of care, offering local tumor control without causing significant local or systemic toxicity (Merle et al. 2017). However, with increasing treatment numbers, it became apparent that the clinical benefit of TACE can be very heterogeneous. While in some patients long-term tumor control can be achieved, in others an almost immediate disease progression is observed. Moreover, it is difficult to predict which patients cope well with the intervention and which develop complications like post-embolization syndrome (PES), a negative predictor for OS itself. Thus, today appropriate selection of patients for TACE is still challenging and controversially debated (Yu 2016; Kudo et al. 2014). In the past, tumor-specific markers (tumor size, numbers and distribution of tumor lesions) in particular have been studied as predictive and/or prognostic factors for patient selection (Yu 2016; Kudo et al. 2014). In addition, the ART (Adhoute et al. 2015; Fatourou and Tsochatzis 2014) or HAP score (Kadalayil et al. 2013) were suggested as novel tools for decision making in patients receiving TACE for HCC. Despite these efforts, no single marker or score for patient selection has yet gained acceptance in routine clinical practice. Therefore, patient selection for TACE is still based on the clinical judgment or expertise of the treating physicians or centers, rather than on objective tools.

The individual body composition of patients has been increasingly recognized as a prognostic but also predictive marker in the context of numerous cancers (Shachar et al. 2016). In patients both with primary and secondary liver malignancies, cachexia turned out as a predictor for the patients prognosis after surgery or ablative treatments (Sun et al. 2018; Nakanishi et al. 2018). However, the impact of the body composition on response rates and general outcome in patients receiving TACE therapy is largely unknown. We, therefore, aimed to evaluate a potential role of different parameters of the individual body composition including the skeletal muscle index (SMI), the median muscular attenuation (MMA) as a surrogate for myosteatosis, the bone mineral density as well as the subcutaneous and visceral fat area as predictors of clinical outcomes in patients receiving transarterial chemoembolization.

## Patients and methods

### Study design

A total of 89 patients undergoing TACE therapy at the Department of Diagnostic and Interventional Radiology at University of Düsseldorf (2011- 2021) were used for the present study (detailed patient characteristics are summarized in Table 1). The study protocol was approved by the local ethics committee and conducted in accordance with the ethical standards laid down in the Declaration of Helsinki.

**Table 1:** Basic characteristics of the study cohort

	Study cohort
TACE patients	89
Sex [%]	
Male–female	68.5–31.5
Age [years, median and range]	69 [23–90]
Hepatic malignancy [%]	
HCC	87.7
Liver metastasis	21.3
Etiology of liver disease (HCC only) [%]:	
Alcoholic	20.0
HBV	14.3
HCV	27.1
NAFLD	8.6
Others	30.0
Size of target lesion [cm, median and range]	divided by the square of the3.3 [0.8–15.0]
OR to TACE therapy [%]	
Yes–no	81.6–18.4
Survival yes–no [%]	
6 months	94.0–6.0
12 months	84.3–15.7
24 months	60.0–40
Median overall survival [days]	1261

*TACE* transarterial chemoembolization, *HCC* hepatocellular carcinoma, *OR* objective response

### Transarterial chemoembolization (TACE)

An emulsion of a chemotherapeutic agent and an embolic agent diluted with iodized contrast (Ultravist 300, Bayer Vital GmbH, Leverkusen, Germany) was used for TACE. For HCCs, in 49 cases, a combination of doxorubicin and spherical, tightly calibrated, biocompatible, non-resorbable, hydrogel microspheres coated with an inorganic perfluorinated polymer (Polyzene™-F, Embozene 100 μm Boston Scientific) was used. In 21 cases, a combination of ethiodized oil (Lipiodol Ultra-Fluid Guerbet France) and doxorubicin 50 mg was used. Liver metastases (*n* = 19) were treated using a chemotherapeutic agent as well as degradable starch microspheres (Embozene 100 μm) or drug eluting beads (DcBeads, BTG International Ltd, London, UK). TACE was performed via the right femoral artery. Hepatography was performed with a microcatheter. Whenever possible, a superselective approach was used.

### Evaluation of TACE response

Within our study, the response to TACE was examined using a multidetector CT scan with multiphasic, contrast-enhanced acquisitions in unenhanced, arterial, portal venous as well as late-venous phase. The last imaging before TACE (no more than 4 weeks old) and the first imaging 6–8 weeks after TACE were used to assess response. All imaging procedures were performed as part of the clinical routine. Response assessment was conducted according to RECIST 1.1 criteria in cases of metastases from gastrointestinal cancers (Eisenhauer et al. 2009) and mRECIST criteria for hepatocellular carcinoma (Lencioni and Llovet 2010). Complete response (CR) and partial response (PR) were defined as an objective response (OR); thus, OR was calculated as the sum of patients showing CR or PR (Edeline et al. 2012).

### Assessment of the body composition

**Fig. 1:**
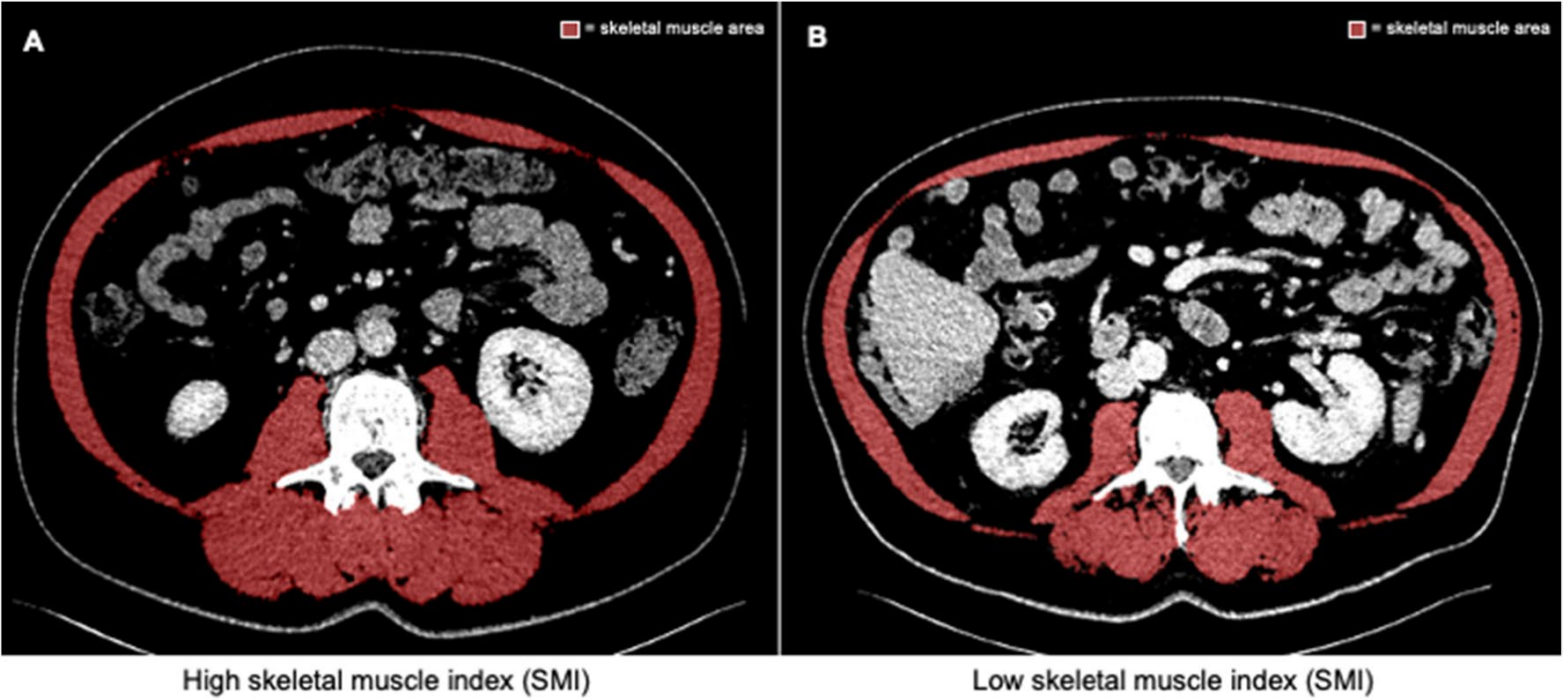
Measurement of the SMI using routine CT scans. (**A**) Example presentation of a patient with a high SMI. **B** Example presentation of a patient with a low SMI

Routine pre-interventional CT scans were used to determine a total of five parameters of the body composition: (1) skeletal muscle index (SMI), (2) median muscular attenuation (MMA, a surrogate for myosteatosis), (3) bone mineral density (BMD), (4) visceral fat area, and (5) subcutaneous fat area. All parameters were measured in venous phase at the level of the third lumbar vertebra in a single slice. The SMI, MMA, visceral fat area, and subcutaneous fat area were measured using the 3D-Slicer tool as previously described (Fedorov et al. 2012). To determine the skeletal muscle area, the muscle areas of the m. psoas, m. erector spinae, m. quadratus lumborum, m. rectus abdominis, m. transversus abdominis, m. obliquus abdominis internus, and m. obliquus abdominis externus were included. Figure 1 displays an exemplary 3D slicer image. To normalize the muscle area for the patient’s height, it was divided by the square of the height. The skeletal muscle index (SMI) was defined as:

$${\text{SMI }}\left[ {{\text{cm}}^{{2}} {\text{/m}}^{{2}} } \right]\, = \,\frac{{{\text{skeletal muscle area at third lumbar vertebra}} \left[ {{\text{cm}}^{2} } \right] }}{{{\text{patients}}^{,} {\text{height}} \left[ {\text{m}} \right]^{2} }}$$

### Statistical analysis

Statistical analyses were performed as described before (Loosen et al. 2017). All statistical analyses were performed with SPSS 23 (SPSS, Chicago, IL, USA) (Koch et al. 2011). A *p *value of < 0.05 was considered statistically significant (*p < 0.05; **p < 0.01; ***p < 0.001).

## Results

### Basic characteristics

We included 89 patients undergoing TACE at our center into analysis (HCC: 70 patients, liver metastases: 19 patients). 68.5% of patients were male and 31.5% were female. The median age was 69 years and ranged from 23 to 90 years. Underlying disease etiologies (HCC only) were distributed as follows: 20% alcoholic, 14.3% HBV, 27.1% HCV, 8.6% non-alcoholic fatty liver disease (NAFLD), 30% others. Table 1 provides a detailed overview of patients’ characteristics.

### Parameters of body composition among different TACE patients

Based on routine CT scans, we evaluated five parameters of the individual body composition in our cohort of TACE patients (see Patients and methods for details): (1) skeletal muscle index (SMI, in cm^2^/m^2^), (2) bone mineral density (BMD, in HU), (3) median muscular attenuation (MMA in HU, a surrogate for myosteatosis), (4) visceral fat area (in cm^2^), (5) subcutaneous fat area (in cm^2^). Median and ranges of the study total cohort are displayed in Tables 2 and 3.

**Table 2:** Parameters of the body composition and laboratory parameters of TACE patients

Parameter (median and range)	Pre-TACE	6 months post TACE
SMI (cm^2^/m^2^)	44.43 (26.97–107.22)	43.51 (14.35–72.37)
Bone mineral density (HU)	139.0 (64.0–467.0)	136.5 (56.0–314.0)
MMA (HU)	35.0 (11.0–61.0)	33.5 (-11.0–62.0)
Visceral fat area (cm^2^)	198.60 (12.94–1598.31)	171.93 (36.88–624.67)
Subcutaneous fat area (cm^2^)	183.39 (45.16–435.06)	157.06 (7.46–474.77)
Potassium (mmol/l)	4.3 (3.2–5.4)	–
Creatinine (mg/dl)	1.00 (0.5–4.63)	–
Urea (mg/dl	33.0 (14.0–134.0)	–
CRP (mg/dl)	0.4 (0.1–17.9)	–
Bilirubin (mg/dl)	0.76 (0.26–6.20)	–
AST (U/l)	39.5 (17.0–346.0)	–
ALT (U/l)	29.0 (6.0–256.0)	–
GGT (U/l)	122.5 (16.0–795)	–
ALP (U/l)	118.0 (51.0–487.0)	–
LDH (U/l)	234.5 (136.0–2131.0)	
Albumin (g/dl)	4.0 (2.8–4.7)	–
WBC (× 1000/µl)	6.2 (1.9–12.9)	–
Hemoglobin (g/dl)	13.0 (8.4–17.3)	–
Platelets (× 1000/µl)	154.5 (34.0–401.0)	–

*SMI* skeletal muscle index, *MMA* median muscular attenuation, *AST* aspartate transaminase, *ALT* alanine transaminase, *GGT* gamma-glutamyl transferase, *ALP* alkaline phosphatase, *CRP* C-reactive protein, *LDH* lactate dehydrogenase, *WBC* white blood cell count

**Table 3:** Univariate and multivariate Cox regression analysis for the prediction of overall survival

Parameter	Univariate Cox regression		Multivariate Cox regression	
	*p* value	Hazard ratio (95% CI)	*p* value	Hazard ratio (95% CI)
SMI	0.001	0.929 (0.888–0.971)	0.014	0.899 (0.827–0.979)
Bone mineral density	0.861	1.000 (0.995–1.004)		
MMA	0.388	0.985 (0.952–1.019)		
Visceral fat area	0.366	1.000 (1.000–1.000)		
Subcutaneous fat area	0.716	1.000 (1.000–1.000)		
Size of target lesion	0.940	1.004 (0.898–1.123)		
Age	0.839	1.003 (0.974–1.032)		
Sex	0.053	0.495 (0.242–1.010)	0.536	0.682 (0.203–2.292)
Creatinine	0.296	0.484 (0.124–1.887)		
AST	0.282	1.003 (0.997–1.010)		
ALT	0.913	0.999 (0.989–1.010)		
GGT	0.742	1.000 (0.998–1.002)		
ALP	0.498	1.002 (0.997–1.006)		
CRP	0.142	1.101 (0.968–1.252)	0.788	0.943 (0.614–1.447)
Bilirubin	0.072	0.521 (0.256–1.059)	0.526	0.766 (0.336–1.745)
LDH	0.001	1.002 (1.001–1.004)	0.001	1.003 (1.001–1.004)
Potassium	0.768	0.870 (0.345–2.196)		
WBC	0.428	1.074 (0.900–1.281)		
Hemoglobin	0.053	0.813 (0.659–1.003)	0.276	0.850 (0.635–1.138)
Platelets	0.514	1.001 (0.997–1.005)		

*SMI* skeletal muscle index, *MMA* median muscular attenuation, *AST* aspartate transaminase, *ALT* alanine transaminase, *GGT* gamma-glutamyl transferase, *ALP* alkaline phosphatase, *CRP* C-reactive protein, *LDH* lactate dehydrogenase, *WBC* white blood cell count

**Fig. 2:**
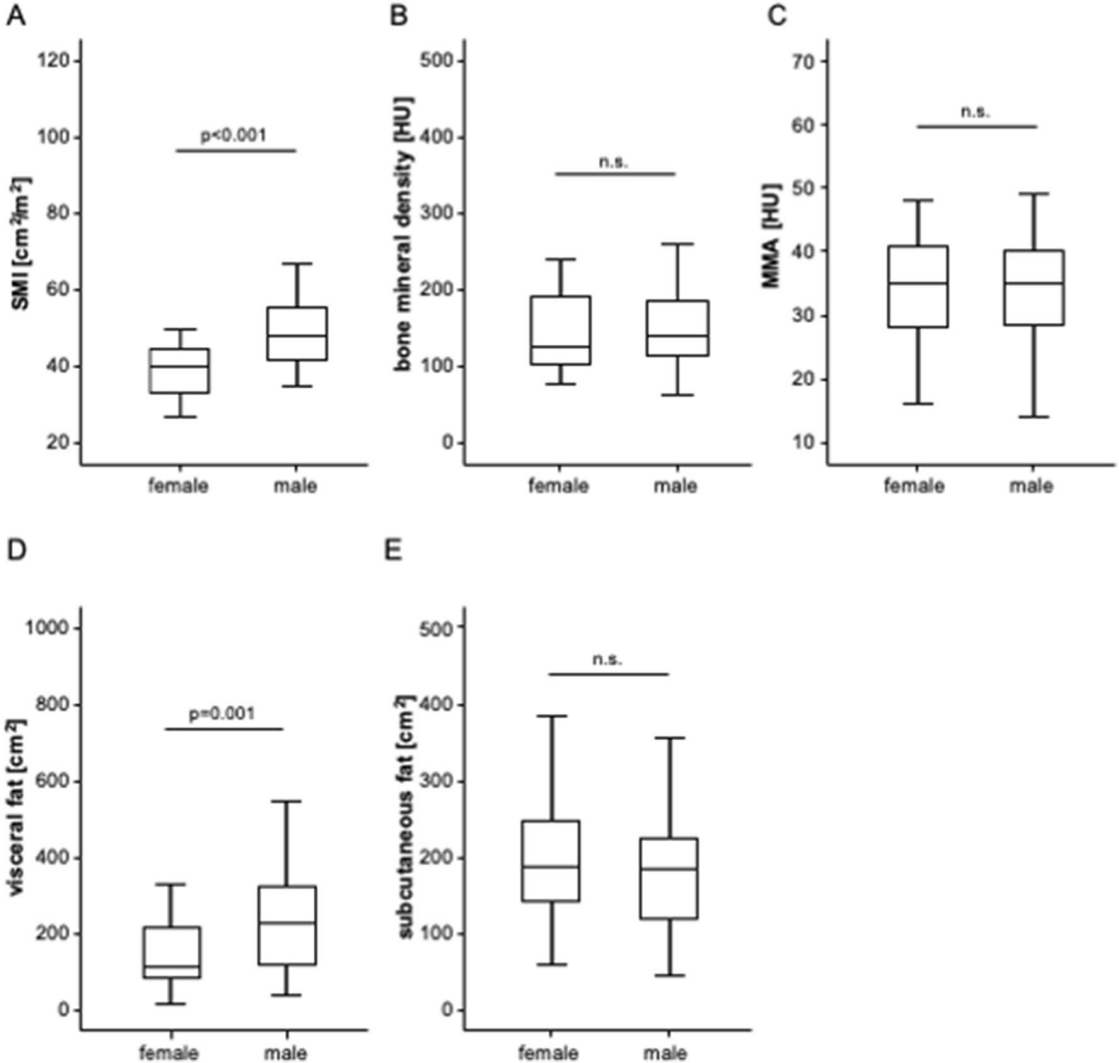
Parameters of the body composition in male and female patients. **A** The SMI and visceral fat area **D** are significantly higher in male patients compared to female patients. **B**, **C,** and **E** The bone mineral density, MMA, and the subcutaneous fat are unaltered between the sexes

We first evaluated potential differences of the body composition between patients with primary and secondary liver cancer. Interestingly, we observed significantly lower SMI and visceral fat area values among patients with liver metastases compared to HCC patients (Suppl. Figure 1A and D). BMD, MMA, and the subcutaneous fat area were comparable between patients with HCC and liver metastases (Suppl. Figure 1B, C and E). In terms of patients’ sex, the SMI and visceral fat area were significantly higher in male patients compared to female patients (Fig. 2A, D), while the other parameters did not significantly differ between sexes (Fig. 2B, C and E). Finally, SMI, BMD as well as visceral or abdominal fat area were not significantly altered in patients with chronic liver disease caused by alcoholic hepatitis, HBV, HCV, and others (HCC only, Suppl. Figure 2A–E). Only MMA was significantly higher among patients with HBV or and HCV (Suppl. Figure 2C).

### Predictive relevance of the individual body composition with respect to TACE response rates

**Fig. 3:**
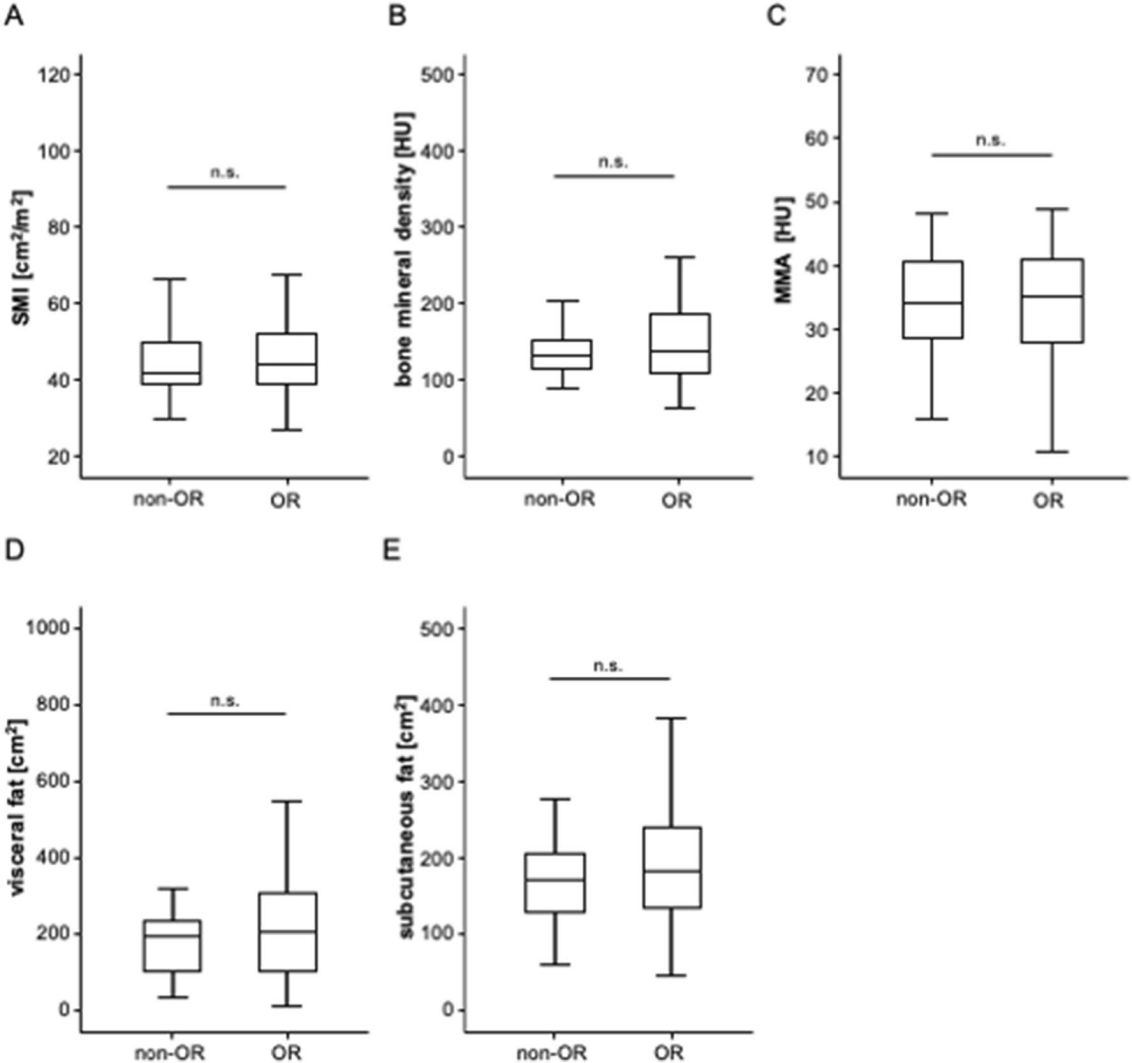
Parameters of the body composition and response to TACE therapy. There are no significant differences regarding the SMI (**A**), bone mineral density (**B**), MMA (**C**), visceral fat area (**D**) or subcutaneous fat area (**E**) between patients who did or did not show an OR to TACE

In the next step, we evaluated whether the individual body composition might have a predictive role with respect to TACE response rates. An objective tumor response (OR, including partial response and complete response) was observed in 81.6% of patients (see Patients and methods for details). Comparing pre-interventional parameters of body composition between patients who did or did not show OR to TACE, we did not observe significant differences regarding the SMI, BMD, MMA, visceral fat area or subcutaneous fat area (Fig. 3A–E). In line, binary logistic regression analysis did not reveal a predictive relevance for an OR to TACE for SMI (Odds ratio (OR): 1.014, 95% CI 0.964–1.067), BMD (OR: 1.003, 95% CI 0.993–1.014), MMA (OR: 0.998, 95% CI 0.943–1.056), as well as the visceral (OR: 1.000, 95% CI 1.000–1.000) or subcutaneous fat area (OR: 1.000, 95%CI: 1.000–1.000).

### SMI predicts long-term survival in patients receiving transarterial chemoembolization

**Fig. 4:**
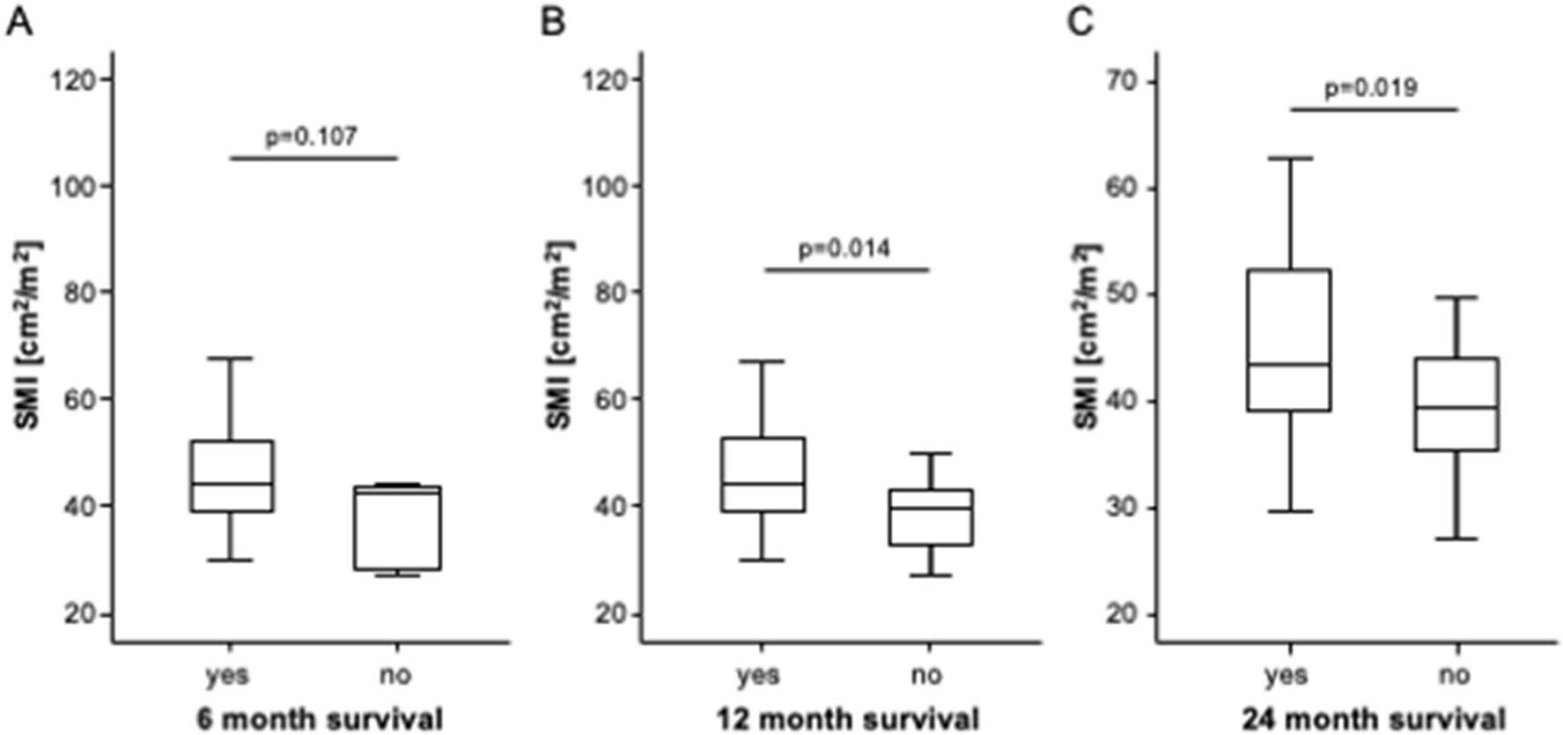
The SMI is a predictor of mortality following TACE therapy. The SMI shows a trend toward lower values in patients who died within 6 months (**A**) and is significantly lower in patients who died within the first 12 (**B**) or 24 (**C**) months after TACE compared to survivors, respectively

We subsequently evaluated a potential prognostic relevance of the pre-interventional body composition on short- and long-term mortality of patients undergoing TACE for primary or secondary liver cancer. We first compared the pre-interventional parameters of the body composition between patients died within 6, 12, and 24 months following TACE and survivors. The SMI was significantly lower in patients who died within the first 12 or 24 months after TACE and showed a trend toward lower values in patients who died within 6 months compared to surviving patients, respectively (Fig. 4A–C). In contrast, there were no significant differences of BMD (*p*_6months_: 0.891, *p*_12months_: 0.690, and *p*_24month_: 0.924), MMA (*p*_6months_: 0.156, *p*_12months_: 0.480, and *p*_24month_: 0.539), visceral fat area (*p*_6months_: 0.904, *p*_12months_: 0.623, and *p*_24month_: 0.452) or subcutaneous fat area (*p*_6months_: 0.774, p_12months_: 0.790, and *p*_24month_: 0.488) between patients who did or did not survive for 6, 12, and 24 months respectively.

**Fig. 5:**
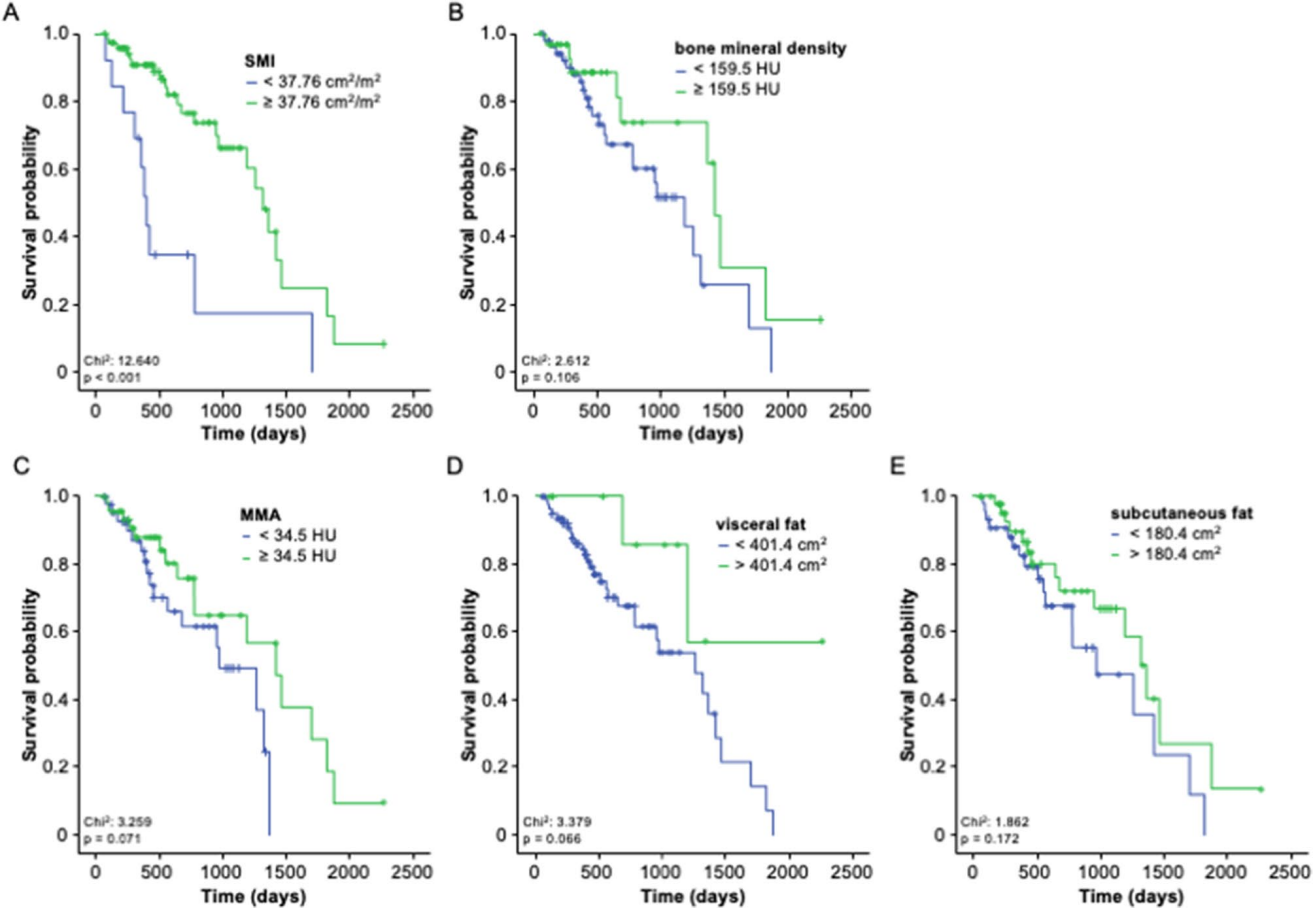
A low SMI is a prognostic factor for OS after TACE therapy. **A** Patients with a pre-interventional SMI below the ideal cutoff value of 37.76 cm^2^/m^2^ have a significantly reduced median OS compared to patients with a SMI above the optimal cutoff value. **B**–**E** No significant differences in long-term survival are observed for the other parameters of the body composition

In a second step, we performed Kaplan–Meier curve analysis to estimate the impact of the body composition on overall survival (OS). When using the median value (50^th^ percentile) of the respective body composition parameter as a cutoff value, we observed a significantly reduced OS in patients who had a pre-interventional SMI below the median of 44.43 cm^2^/m^2^ compared to patients with a SMI value above the cutoff (*p* = 0.004, Suppl. Figure 2A). In contrast, there was no difference in OS between patients with a high or low BMD (Fig. 5B), MMA (Fig. 5C), and visceral or subcutaneous fat area (Fig. 5D, E). We subsequently established ideal prognostic cutoff values as recently described (Budczies et al. 2012). Using these ideal cutoff values, the prognostic relevance of the SMI further increased. Patients with a pre-interventional SMI below 37.76 cm^2^/m^2^ had a significantly reduced median OS of only 404 days compared to a median OS of 1321 days among patients with a SMI above this optimal cutoff value (Fig. 5A, *p* < 0.001). Although trends toward an impaired post-interventional OS in patients with a low MMA (*p* = 0.071) or a low visceral fat area (*p* = 0.066) became apparent when using the respective ideal cutoff values, no significant differences in long-term survival were observed for the other parameters of the body composition (Fig. 5B–E).

The prognostic relevance of the patients’ pre-interventional body composition was finally evaluated in univariate and multivariate Cox regression models. In univariate analyses, the SMI but not the other parameters of the body composition showed a prognostic relevance for OS (Hazard ratio (HR): 0.929, 95% CI 0.888–0.971, *p* = 0.001, Table 2). When including all clinical and laboratory parameters with a *p* value < 0.150 (sex, CRP, bilirubin, LDH, hemoglobin) into a multivariate model, the SMI turned out as an independent prognostic parameter for OS following TACE (HR: 0.899, 95% CI 0.827–0.979, *p* = 0.014, Table 2).

## Discussion

By analyzing a panel of five different parameters reflecting the patient’s body composition in a cohort of patients receiving transarterial chemoembolization for HCC or liver metastases from a gastrointestinal cancer, we show that a low skeletal muscle mass represents a negative prognostic parameter for these patients. In contrast, the bone mineral density, muscle quality, visceral adipose tissue, and subcutaneous fat tissue were not predictive for the patients´ prognosis. Interestingly, despite reflecting the patient’s prognosis, SMI was not indicative for the tumor response toward TACE.

Recently, the topic of body composition has received increasing attention in the context of cancer research. Body composition is defined by *“*the distribution of body mass between separate compartments: fat-free tissue or lean body mass, extracellular water, and adipose tissue*”* (Withrow and Vail 2007). Thus, analyses of the body composition are methods to define different tissue composition of the human body comprising assessment of bone mass and quality, of the muscle and different fat tissue areas (Yeung et al. 2019; Dunne et al. 2019; Albano et al. 2019; Kim et al. 2017). Different methods have been proposed for this purpose in the past, but many of these are complex and/ or not available in routine clinical practice. In contrast, computed tomography (CT) allows to determine all aspects of the body composition as a by-product of the staging procedure performed as part of the routine clinical patients´ management before any therapeutic intervention (Meyer et al. 2022). Such easy-accessible data might be of tremendous clinical importance. Multimodal treatment strategies were recently introduced into clinical algorithms for patients with liver cancer (Finn et al. 2017; Kulik et al. 2017). As an example, TACE is currently used both in neoadjuvant and palliative settings (Park et al. 2015; Massmann et al. 2015; Sacco et al. 2017). Therefore, identifying those patients that have an optimal benefit from this more and more important treatment modality would tremendously improve the clinical management of many patients. Here, we demonstrate that low skeletal muscle mass, as a surrogate for the presence of sarcopenia predicts the patients´ post-interventional prognosis. Patients with an SMI below the ideal cutoff value of 37.76cm^2^/m^2^ had a reduced long-term survival (404 d) compared to patients with a high SMI (1321 d). In contrast to SMI, any other marker for the patients´ body composition was indicative for the patients´ prognosis at least in our cohort. These data surprise at first glance, since it seemed likely that, for example, poor muscle quality (MWA), and high rates of visceral fat would be present in those patients that also display a low SMI. That this assumption did not prove true indicates that the respective markers reflect very different aspects and cannot be interchanged one-to-one, highlighting the need for further pathophysiological research in such patients.

Both sarcopenia and cachexia represent inflammatory conditions (Xie et al. 2022; Malla et al. 2022). We, therefore, hypothesized that patients with improvement in their cachexia have a better prognosis than patients whose cachexia remains the same or even increases over the course of treatment. We next analyzed whether longitudinal change of the SMI is predictive for the patients’ outcome. Strikingly, in these analyses, the individual difference in SMI (deltaSMI) did not affect the patients´ prognosis. These results are surprising, as we and other groups have recently published contrary results (Loosen et al. 2019; Zhang et al. 2022). From these data as well as general clinical considerations, we had recently concluded that: “parameters for sarcopenia should not only be integrated into algorithms for the clinical decision making in patients eligible for TACE but might also be used to trigger specific measures in terms of nutritional support*”* (Loosen et al. 2019*).* Although in the present analysis, we could not confirm our recent findings on deltaSMI as a prognostic marker, we remain convinced that such measures must be a critical component in the clinical management of patients with TACE, which is in line to current recommendations for the clinical management of patients with liver cancer (Galle et al. 2018). Nevertheless, our current data challenge the previous findings, and therefore support the need for large and randomized trials to finally prove the effect of nutritional interventions in patients with cancer and in particular in cancer patients undergoing TACE.

The presented data highlight the role of muscle mass determined before TACE as a parameter for stratifying patients according to their individual prognosis. The easy availability and cost effectiveness make SMI an attractive parameter that could be easily integrated into existing algorithms for prognostic assessment of TACE patients. Nevertheless, our analyses face important limitations, which are due to the study design and cannot be avoided. First, our study included only 89 patients representing a rather small cohort of patients when analyzing complex endpoints such as overall survival. Second, our study represents a retrospective analysis conducted at a single center only, and thus center-specific bias cannot be excluded. Moreover, patients were included over a very long period of time during which the specific methodology of TACE has changed. In addition, patients with several different tumor entities were included. Therefore, we cannot exclude that the mixing of effects may have shifted the final results of the study in one direction or the other. Finally, our data do not provide evidence on whether an individual patient should have received another treatment strategy than TACE. This important clinical question can only be answered by further prospective clinical studies including different treatment modalities. Such studies would not only improve the clinical management of patients with primary and secondary liver tumors but could also provide important insights into the pathophysiology of sarcopenia in cancer patients if appropriate translational programs are integrated into the respective study design.

## Supplementary Information

Below is the link to the electronic supplementary material.

Supplementary file1 Parameters of the body composition in patients with HCC and liver metastases (A) The skeletal muscle index (SMI) is significantly lower in patients with liver metastases compared to HCC patients. (B, C) The bone mineral density and MMA are comparable between patients with HCC and liver metastases. (D) The visceral fat area is significantly lower in patients with liver metastases compared to HCC patients. (E) There is no difference of the subcutaneous fat area between patients with HCC and liver metastases (TIFF 1054 KB)

Supplementary file2 The SMI (A), bone mineral density (B) as well as the visceral (D) or abdominal (E) fat area are not significantly altered in patients with chronic liver disease caused by alcoholic hepatitis, HBV, HCV and others. (C) The MMA is significantly higher among patients with HBV or and HCV (TIFF 1054 KB)

Supplementary file3 (A) Patients with a pre-interventional SMI below the 50th percentile have a significantly reduced median OS compared to patients with a SMI above the optimal cut-off value. (B-E) No significant differences in long-term survival are observed for the other parameters of the body composition (TIFF 1054 KB)

## Data Availability

Data are available from the corresponding author upon meaningful request.
