# Supplementary figures and images for: Sarcopenia indicate poor survival in patients undergoing transarterial chemoembolization (TACE) for hepatic malignancies
Source: J Cancer Res Clin Oncol. 2023 Jan 23;149(9):6181–90. doi: 10.1007/s00432-022-04519-8 (PMC10356883; doi:10.1007/s00432-022-04519-8)

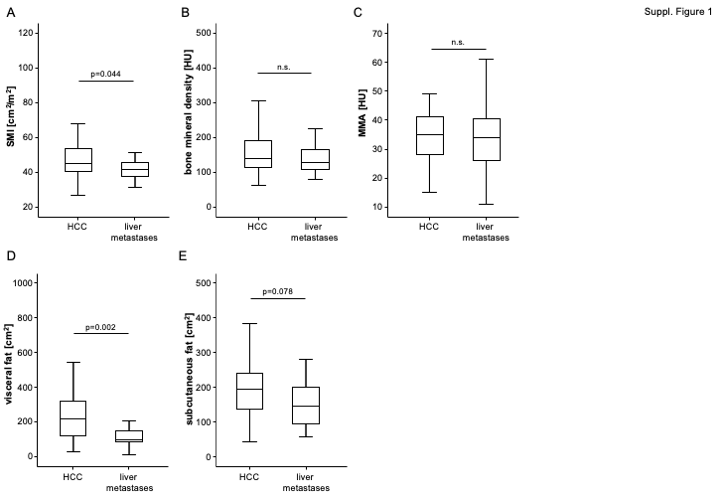

Supplement: Supplementary file 1 — Supplementary file1 Parameters of the body composition in patients with HCC and liver metastases (A) The skeletal muscle index (SMI) is significantly lower in patients with liver metastases compared to HCC patients. (B, C) The bone mineral density and MMA are comparable between patients with HCC and liver metastases. (D) The visceral fat area is significantly lower in patients with liver metastases compared to HCC patients. (E) There is no difference of the subcutaneous fat area between patients with HCC and liver metastases (TIFF 1054 KB) [file 432_2022_4519_MOESM1_ESM.tiff]

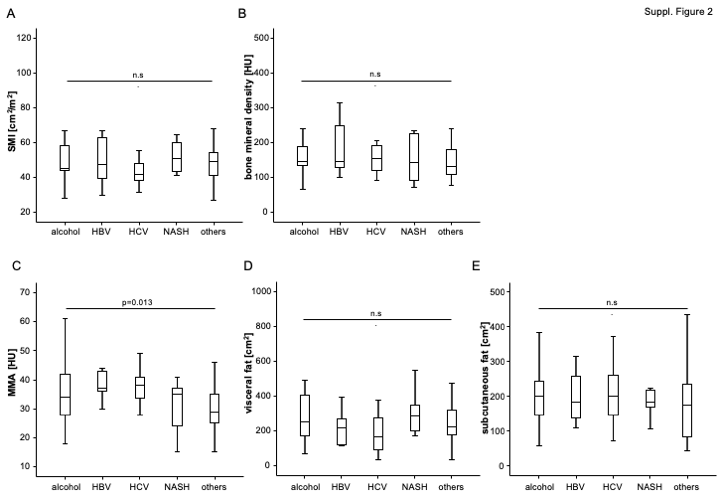

Supplement: Supplementary file 2 — Supplementary file2 The SMI (A), bone mineral density (B) as well as the visceral (D) or abdominal (E) fat area are not significantly altered in patients with chronic liver disease caused by alcoholic hepatitis, HBV, HCV and others. (C) The MMA is significantly higher among patients with HBV or and HCV (TIFF 1054 KB) [file 432_2022_4519_MOESM2_ESM.tiff]

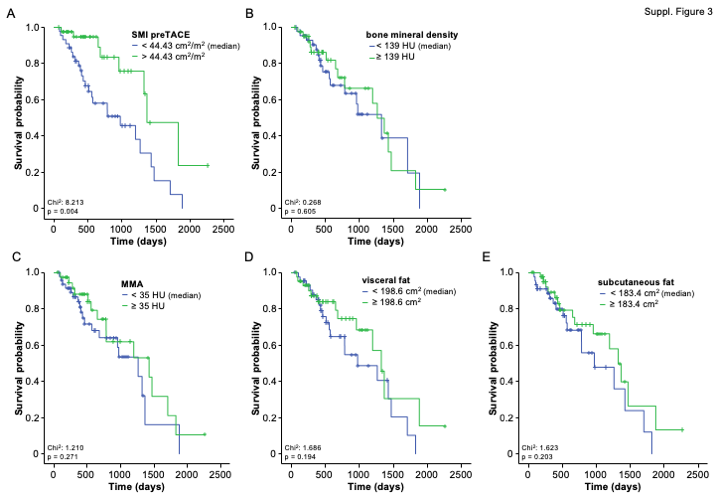

Supplement: Supplementary file 3 — Supplementary file3 (A) Patients with a pre-interventional SMI below the 50th percentile have a significantly reduced median OS compared to patients with a SMI above the optimal cut-off value. (B-E) No significant differences in long-term survival are observed for the other parameters of the body composition (TIFF 1054 KB) [file 432_2022_4519_MOESM3_ESM.tiff]
